# Visualizing Cell State Transition Using Raman Spectroscopy

**DOI:** 10.1371/journal.pone.0084478

**Published:** 2014-01-07

**Authors:** Taro Ichimura, Liang-da Chiu, Katsumasa Fujita, Satoshi Kawata, Tomonobu M. Watanabe, Toshio Yanagida, Hideaki Fujita

**Affiliations:** 1 Quantitative Biology Center, Riken, Suita, Osaka, Japan; 2 Department of Applied Physics, Osaka University, Suita, Osaka, Japan; 3 Nanophotonics Laboratory, Riken, Wako, Saitama, Japan; 4 Immunology Frontier Research Center, Osaka University, Suita, Osaka, Japan; Dalhousie University, Canada

## Abstract

System level understanding of the cell requires detailed description of the cell state, which is often characterized by the expression levels of proteins. However, understanding the cell state requires comprehensive information of the cell, which is usually obtained from a large number of cells and their disruption. In this study, we used Raman spectroscopy, which can report changes in the cell state without introducing any label, as a non-invasive method with single cell capability. Significant differences in Raman spectra were observed at the levels of both the cytosol and nucleus in different cell-lines from mouse, indicating that Raman spectra reflect differences in the cell state. Difference in cell state was observed before and after the induction of differentiation in neuroblastoma and adipocytes, showing that Raman spectra can detect subtle changes in the cell state. Cell state transitions during embryonic stem cell (ESC) differentiation were visualized when Raman spectroscopy was coupled with principal component analysis (PCA), which showed gradual transition in the cell states during differentiation. Detailed analysis showed that the diversity between cells are large in undifferentiated ESC and in mesenchymal stem cells compared with terminally differentiated cells, implying that the cell state in stem cells stochastically fluctuates during the self-renewal process. The present study strongly indicates that Raman spectral morphology, in combination with PCA, can be used to establish cells' fingerprints, which can be useful for distinguishing and identifying different cellular states.

## Introduction

Systems biology is a field of science to understand the biological system's network structure and dynamics rather than just characterizing the function of isolated parts [1]. Advances in computational power and algorithms have pushed systems biology into a new era, enabling to simulate a life of a small organism *in silico* [2]. Stem cell self renewal and differentiation are attractive targets for systems biology owing to their importance in the life sciences. Systems-level understanding of complex biological system, such as the gene regulatory networks of ESCs, requires comprehensive knowledge of the components and their interactions within a single ESC. However, advances in measurement technology have not yet realized the acquisition of such comprehensive data at the single cell level. As it stands, current systems biology approaches for ESCs thus deals with a limited number of transcription factor networks including the core pluripotency factors [3], [4], restricting the understanding of the complicated transcriptional network of ESC.

Another approach to understand self renewal and differentiation of ESC is to grasp the changes in the complicated network as whole and visualizing the state transitions on a cell-state landscape. This idea was first introduced by Waddington, where the differentiation potential was drawn as an epigenetic landscape [5], in which the differentiation process is represented as cells rolling down the potential. This type of approach does not necessarily require comprehensive analysis, but often needs quantitative estimations. For example, the cell state can be often estimated by the morphology of the cell, which is also the case in ESC where undifferentiated ESCs form highly packed colonies. Thus, as far as the indices reflect the internal state of the cell, it can be used to describe the state transition of the cell, and accumulated paths of state transition observed in single cell will draw the cell-state landscape.

To this end, we focus on Raman scattering microscopy to obtain information of the cell state at the single cell level. The Raman scattering phenomenon arises from molecular vibrations, providing information on chemical species, composition, and the amount of constituent molecules. Thus, Raman scattering imaging can simultaneously detect the location and amount of multiple compounds such as proteins, lipids, DNA, and RNA [6]. Recent advances in Raman scattering microscopy have pushed its applicability to the investigation of biological phenomena in medical and clinical assays for which non-invasive methods are required [7], [8]. Since the amount and distribution of the intracellular compounds are related to the cell state, Raman microscopy has been used to describe cell states transitions such as apoptosis, differentiation, and cell division, possibly without harming the cells [9], [10], [11]. Furthermore, Raman spectroscopy was used to monitor cell state changes after drug exposure [12], during cell cycle [13], and cell differentiation during embryo development [14], showing the capability of Raman spectroscopy in cell state monitoring.

In this study, to understand the cell state transition during the differentiation, we performed Raman spectral imaging of mouse ESCs during differentiation, and compared the results with those from terminally differentiated cell-lines including fibroblasts, epithelial cells, and hepatocytes. In addition, cells with differentiation capability, such as bone marrow mesenchymal stem cells (MSCs), adipocytes, and neuroblasts, were analyzed which showed significant changes in the Raman spectra during the differentiation process. By carefully analyzing the Raman spectra of the cellular nucleus, we were able to illustrate the differentiation pathway of the ESCs. In this study, we did not concentrate on deducing the molecular species from Raman spectra but we rather tried to interpret the spectral shapes in a morphological way. Here we propose Raman spectrum morphology, a method for visually understanding the cell state without recurring to labeling strategies, and demonstrate the discrimination of the cell state transition on the landscape by Raman spectra.

## Materials and Methods

### Cell culture

Mouse ESCs (E14Tg2a) were purchased from the Riken Cell Bank (Ibaraki, Japan) and maintained on feeder-free gelatin-coated plates in Leukemia Inhibitory Factor (LIF)-supplemented medium: Dulbecco's modified Eagle's medium-High Glucose (DMEM-HG; Invitrogen, Carlsbad, CA) was supplemented with 10% Fetal Bovine Serum (FBS) (Invitrogen), antibiotics (100 U/mL penicillin, 0.1 mg/mL streptomycin) (Invitrogen), 1× GlutaMAX-1 (Invitrogen), 1% 2-mercaptoethanol (Bio-Rad), 1× nonessential amino acids (NEAA) (Invitrogen), and 1 mM sodium pyruvate (Sigma-Aldrich, St. Louis, MO). EPH4 were cultured in 50% DMEM-HG and 50% Ham's F-12 mixture (Wako, Osaka, Japan) supplemented with 1% l-Glutamine (Sigma-Aldrich), 10% FBS (Invitrogen) and antibiotics. Hepa1-6 cells were cultured in DMEM-HG supplemented with 10% Fetal Calf Serum (FCS) and antibiotics. Neuro2a cells were cultured in DMEM-HG supplemented with 10% FBS, 1% NEAA, and antibiotics. For the induction of differentiation, the medium was replaced with DMEM-HG supplemented with 2% FBS, 1% NEAA, and antibiotics, and the cells were cultured for 3 days. 3T3L1 cells were cultured in DMEM-HG supplemented with 10% calf serum (Sigma-Aldrich) and antibiotics. For the induction of differentiation, cells were cultured until confluence. Then, the medium was replaced with DMEM-HG supplemented with 10% FBS, 500 µM isobutylmethylxanthine, 25 µM dexamethasone, 4 µg/mL insulin and antibiotics and cultured for 4 days. Cells were further cultured in DMEM-HG supplemented with 10% FBS, 4 µg/mL insulin and antibiotics for 2 days for maturation.

### Microscopy

For Raman imaging, cells were plated on silica coverslip (SPI supplies, West Chester, PA) coated with either 100 µg/ml E-cadherin (ESC) or 0.1% gelatin (other cells) and cultured for 3 days. For Neuro2a cells, the differentiation was induced directly on the silica coverslip, while 3T3L1 cells were re-plated on the silica coverslip after the differentiation. Just before the observation, the medium was replaced with Tyrode's solution. All data were recorded with a home-built slit-scanning Raman microscope at 532 nm excitation wavelength [11]. A NIKON CFI Plan Apo IR 60× water immersion lens with 1.27 numerical aperture (NA) was used as the objective lens. Perfect Focus System remained active during all measurements. The images were scanned over a 40 µm range and divided into 120 lines. The exposure time of each line was 5 s, and the laser intensity was 2.4 mW/µm^2^.

### Data analysis

PCA was employed for spectral characterization. PCA reduces the dimensionality of a set of spectral data, which corresponds to the wavenumber division of the spectral data, into a few uncorrelated variables called principal components (PCs). During the pre-treatment step, the measured Raman spectra (**x** *_i_*) are first processed as described below and decomposed into linearly uncorrelated loading vectors of the principal components (**p** *_k_*) as follows, 
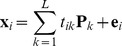
 where t*_ik_* and **e** *_i_* are the scores of the *k*-th principal component for the *i*-th sample and the residual component attributed to noise, and *L* is the number of effective principal components. The loading vectors make an orthonormal coordinate system with a dimension much smaller than the original spectral data. For the extraction of the scores and the loading vectors, we adopted the non-linear iterative partial least squares (NIPALS) algorithm [15].

As a pre-treatment for PCA, we standardized the original spectral data by subtracting the mean value from each spectrum and dividing by its standard deviation [15]. The pre-processed spectral data substituted the original data in the above equation. The standardization process is effective at eliminating both the additive and multiplicative differences of the spectral baseline caused by slight discrepancies in the experimental conditions.

## Results

### Raman spectra of established cell-lines

**Figure 1:**
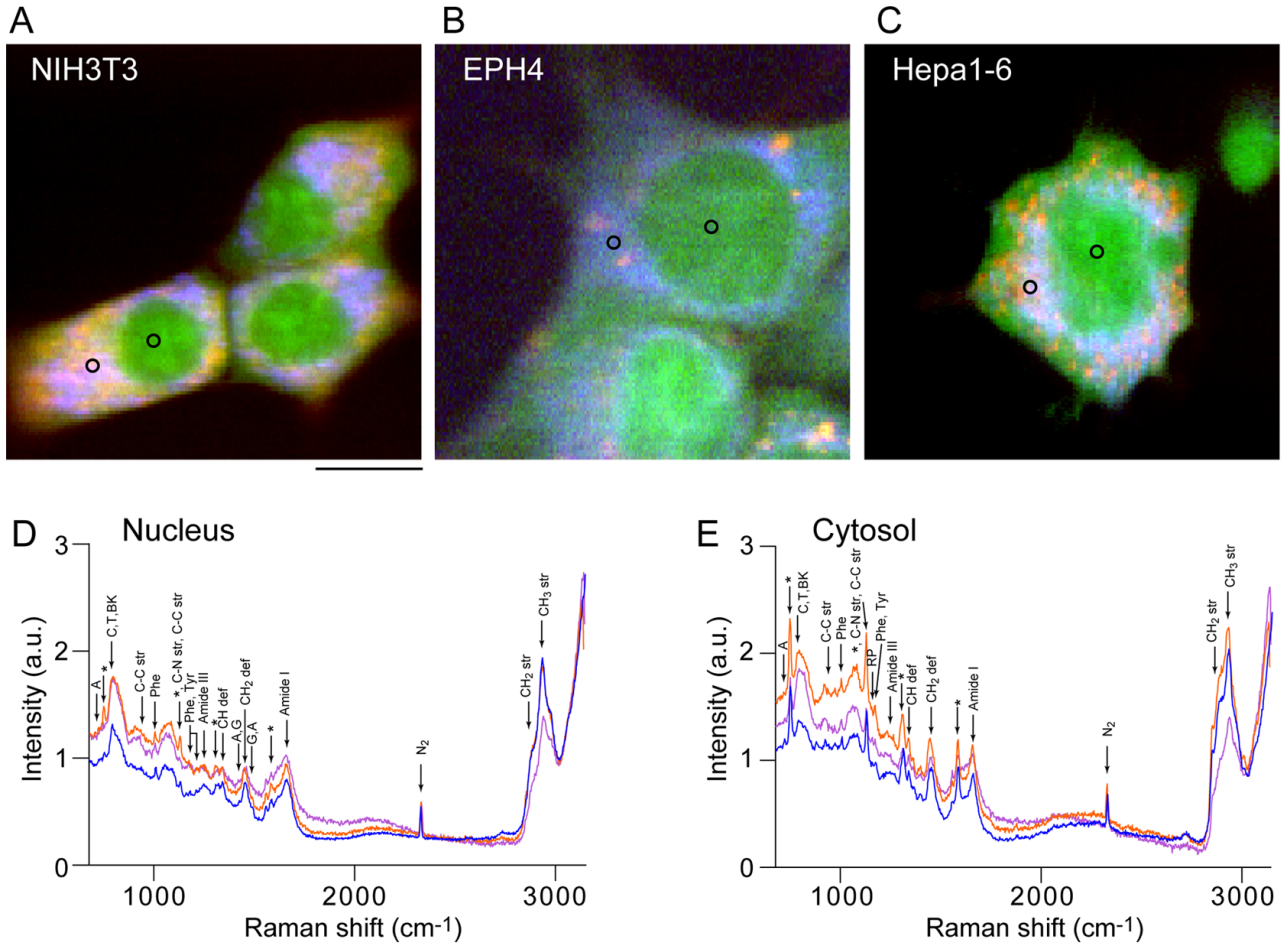
Raman images of three cell-lines. RGB reconstituted Raman images of NIH3T3 (A), EPH4 (B) and Hepa1–6 (C) cells. Raman peaks at 753 cm^−1^ (cytochrome C), 1686 cm^−1^ (proteins), and 2852 cm^−1^ (lipids) are mapped in blue, green, and red, respectively. Scale bar, 10 µm. (D) Raman spectra of the nuclei of NIH3T3 (blue), EPH4 (purple) and Hepa1-6 (orange) cells. (E) Raman spectra of the cytosol of NIH3T3 (blue), EPH4 (purple) and Hepa1-6 (orange). The representative Raman spectra shown are the average of spectra at 49 pixel positions in the black circled region in A–C. Peak assignments are, RP; rebose-phosphate, BK; backbone OPO of nucleic acid, str; stretching, def; deformation [31]. Peaks characteristic to cytochrome C are indicated with asterisks.

Although many types of cells have been analyzed by Raman spectroscopy, it is still uncertain whether cell state can be distinguished by difference in Raman spectra. To clarify whether cell state can be distinguished by Raman spectra, we performed Raman spectral imaging against three cell-lines established from mouse. A home-built Raman microscope [6], [11] was used to observe the cells, and Raman spectra were recorded at all pixel positions (See Methods for details). Our Raman microscopy employs a line confocal scanning method, not point scanning, because of the shorter image acquisition time, which contributes to the improvement of the cell viability after the observation. We used NIH3T3, EPH4 and Hepa1–6 cells as models for fibroblast, epithelial and hepatocyte cells, respectively [16], [17]. Figure 1A, 1B and 1C is an Red/Green/Blue (RGB) reconstruction of the Raman spectral image, where different RGB colors are assigned to the peaks intensities at 753 cm^−1^ (pyrrole ring breathing mode in cytochrome C; blue), 1660 cm^−1^ (amide I vibration mode mainly in peptide bonds; green), and 2852 cm^−1^ (CH_2_ stretching mode mainly in lipids; red). As opposed to NIH3T3 and EPH4 cells, the Raman images of Hepa1-6 cells showed well-developed mitochondria and an accumulation of lipid droplets (Fig. 1C).

**Figure 2:**
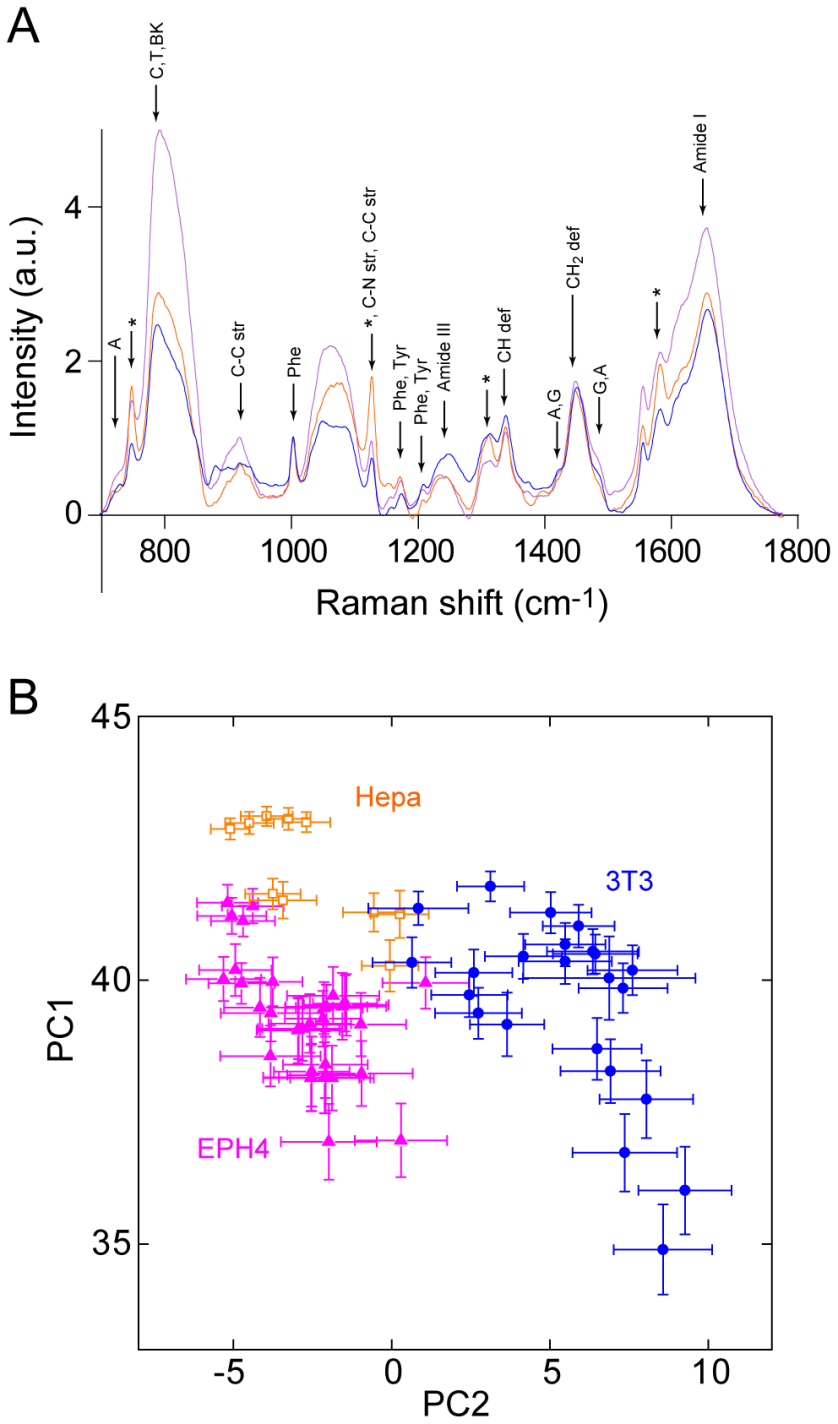
Difference in Raman spectra between cell-lines. (A) Averaged Raman spectra of NIH3T3 (blue), EPH4 (purple) and Hepa1-6 (orange) cells in the fingerprint region (700–1800 cm^−1^). Raman spectra are average of 10–34 cells for each cell-line. The lower envelope, which was estimated by a 4^th^-order polynomial fitting, was subtracted from all spectra in order to make the spectral differences clearer for comparison [32]. (B) Score plots calculated by PCA for three cell-lines. For PCA analysis, raw spectra without averaging was used. Each symbol represents a single cell. NIH3T3 (blue), EPH4 (purple) and Hepa1-6 (orange).

Figure 1D and 1E show the representative Raman spectra at the nucleus (Fig. 1D) and the cytosol (Fig. 1E) from NIH3T3 (blue), EPH4 (purple) and Hepa1-6 (orange) cells, according to regions marked by black circles in Fig. 1A–C. It is clear that the spectral features of these cell-lines are very different at both the nucleus and the cytosol. Although the differences in the spectra between the cell-lines are larger in the cytosol than in the nucleus, the presence of lipid droplets and the large autofluorescence are expected to hinder detailed analysis. Thus, we focused our efforts on the nucleus. Figure 2A shows averaged Raman spectra of the fingerprint region (700–1800 cm^-1^) of the nucleus, where spectra from 23 (3T3), 34 (EPH4) and 10 (Hepa),cells were averaged. In order to visually recognize the spectral changes, the spectra were processed by subtracting the lower envelope and by normalizing with respect to the value of 1005 cm^−1^ (aromatic amino acid) so that the spectral change can be visually recognized. It is important to note that some peaks originate from silica substrate (Fig. S1) and not from cells. The major differences between the three cell-lines were mainly observed in the peaks at 1583 cm^−1^ (cytochrome C) and 1660 cm^−1^ (amide-I), with the latter being the most emphasized and thus a potential marker for distinguishing the cell type. To obtain more significant information from the collected data, we performed PCA, focusing on the fingerprint region of the Raman spectra. PCA is a multivariate analysis methods widely used for spectral analysis of multiple components samples. PCA decomposes the spectra into a linear combination of loading vectors after extracting the number of independent components. The loading vectors make an orthonormal coordinate system with the deduced dimension, and the scores represent the weight coefficients for the loading vectors [18]. Raw spectra was used for PCA and prior to the PCA, all the spectra in the dataset were preprocessed through the standardization manner, which is mean value subtraction followed by division by standard deviation. Three cell-lines appeared in the different regions on the score plot on the PC1-PC2 plane (Fig. 2B), demonstrating that Raman spectra are significantly different between cell-lines. Because all three cell-lines are derived from mouse, different cell-lines can be considered as cells in different states. Thus, this result show that Raman spectra from cell nucleus can be used to identify differences in cell states.

### Observation of differentiation in cell-lines

**Figure 3:**
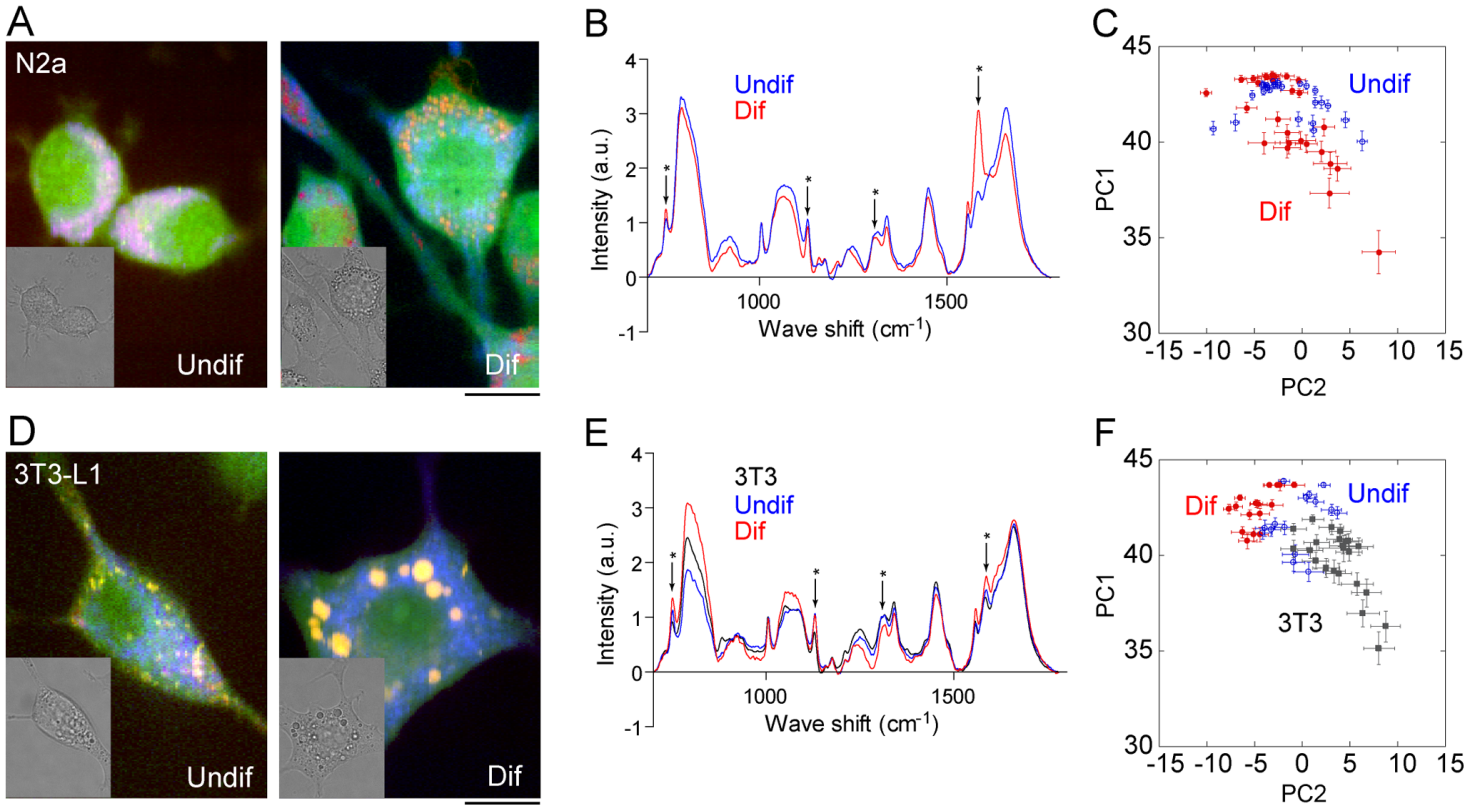
Raman images of cell-lines with differentiation capability. Raman images of Neuro2a (A) and 3T3L1 (D) cells before (left panel) and after (right panel) the induction of differentiation (inset; bright-field image). (B, E) Averaged Raman spectra of N2a (B) and 3T3L1 (E) cells before (blue) and after (red) induction of differentiation. Spectra are average of 15–27 cells. Spectra from the fibroblast cell-line NIH3T3 are also plotted (black). Peaks characteristic to cytochrome C are indicated with asterisks. (C, F) Score plots of Neuro2a (C) and 3T3L1 (F) cells before (blue) and after (red) the induction of differentiation calculated by PCA. For PCA analysis, raw spectra without averaging was used. Data from the fibroblast cell-line NIH3T3 are also plotted (black). Each marker shows averaged score values of the spectra obtained from single nuclei. Error bar shows SD of the score values from the same nuclei.

Although it was shown that Raman spectra from the nucleus can be used to recognize the cell types, it is still uncertain whether small cell state changes such as cell differentiation can be distinguished by Raman microscopy. To investigate whether the Raman imaging can be used to distinguish the differentiation status of the cells, we have performed Raman imaging on cell-lines that are capable of induced differentiation. Neuro2a (N2a) is a mouse neuroblastoma cell-line that is extensively used to study neuronal differentiation [19], while 3T3L1 is a cell-line sub-cloned from NIH3T3 cells that is capable of differentiating into adipocyte-like cells [20]. These cells exhibited characteristic phenotype upon the induction of differentiation, with N2a showing a rapid outgrowth of neurites and 3T3L1 accumulating lipid droplets (Fig. S2). The differentiation status of the cell is often predicted by its morphology, however, changes are small in some cases and is not quantitative. Raman images after differentiation revealed the presence of large lipid droplets and an increased amount of cytochrome C in the cytosol in both cell-lines (Fig. 3A and 3D).

Figure 3B and 3E show averaged Raman spectra from the nucleus before and after the differentiation of N2a (Fig. 3B) and 3T3-L1 (Fig. 3E). Averaged Raman spectra from the nucleus of NIH3T3 cells shown in Fig. 2A is also included in Fig. 3E (black line) for comparison. Large differences can be seen in the Raman spectra following differentiation, especially at 1583 cm^−1^ in both cell-lines. To investigate whether Raman spectra had changed by differentiation, PCA was performed on the two cell-lines separately, with each dataset containing 8–26 nuclei. The score plot on the PC1-PC2 plane shows small but clear differences between the Raman spectra of the differentiated and undifferentiated cells for both N2a and 3T3L1 (Fig. 3C and 3F). This result demonstrates that Raman spectroscopy coupled to PCA can distinguish the differentiation state of the cells.

### Raman spectra of ESCs before and after spontaneous differentiation

**Figure 4:**
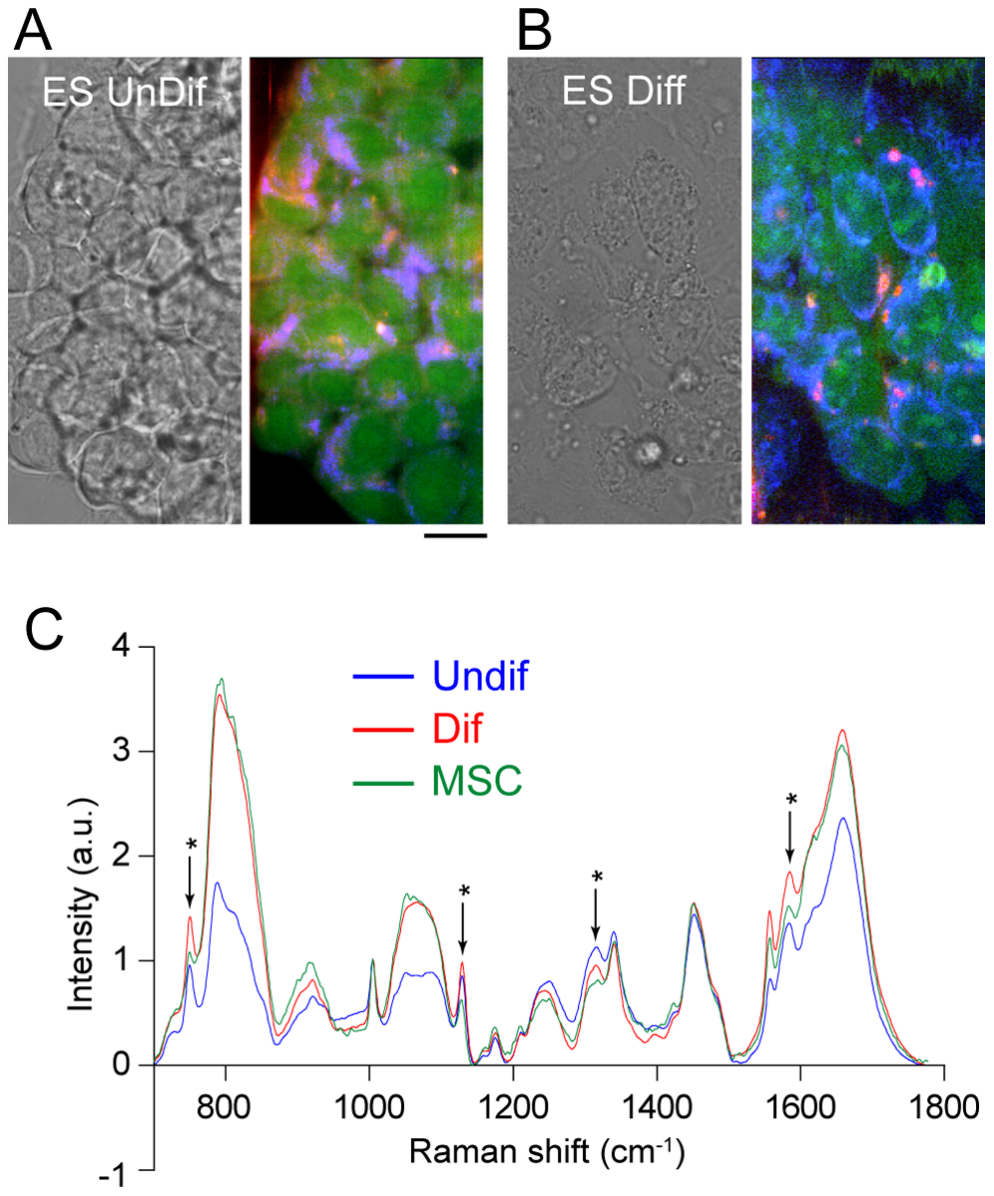
Raman images of ESCs before and after induction of differentiation. Bright-field (left panel) and Raman images (right panel) of undifferentiated (A) and differentiated (B) ESCs. (C) Averaged Raman spectra of undifferentiated ESCs (blue), differentiated ESCs (red), and MSCs (green) in the fingerprint region (700–1800 cm^−1^). For PCA analysis, raw spectra without averaging was used. Spectra shown are average of 18–44 cells.

Although cell-lines are an excellent model for studying the cell state change during differentiation, they are an experimental model and therefore may not reflect the natural cells. Thus, we next tested whether cell state change during differentiation can be detected by Raman imaging in ESCs, which can be considered as a more natural state of the cell. To culture mouse ESCs, silica coverslips were coated with E-cadherin, which enabled the formation of single and/or double layered colonies (Fig. 4A, left panel). Figure 4A (right panel) shows an RGB reconstruction of the Raman spectral image of ESCs before induction of differentiation. Seven days after the removal of the LIF, whose absence is known to induce mouse ESCs differentiation [21], cells became flatter and spread over the substrate. At the same time, their Raman image showed the presence of lipid droplets, which are absent in undifferentiated cells (Fig. 4B).

**Figure 5:**
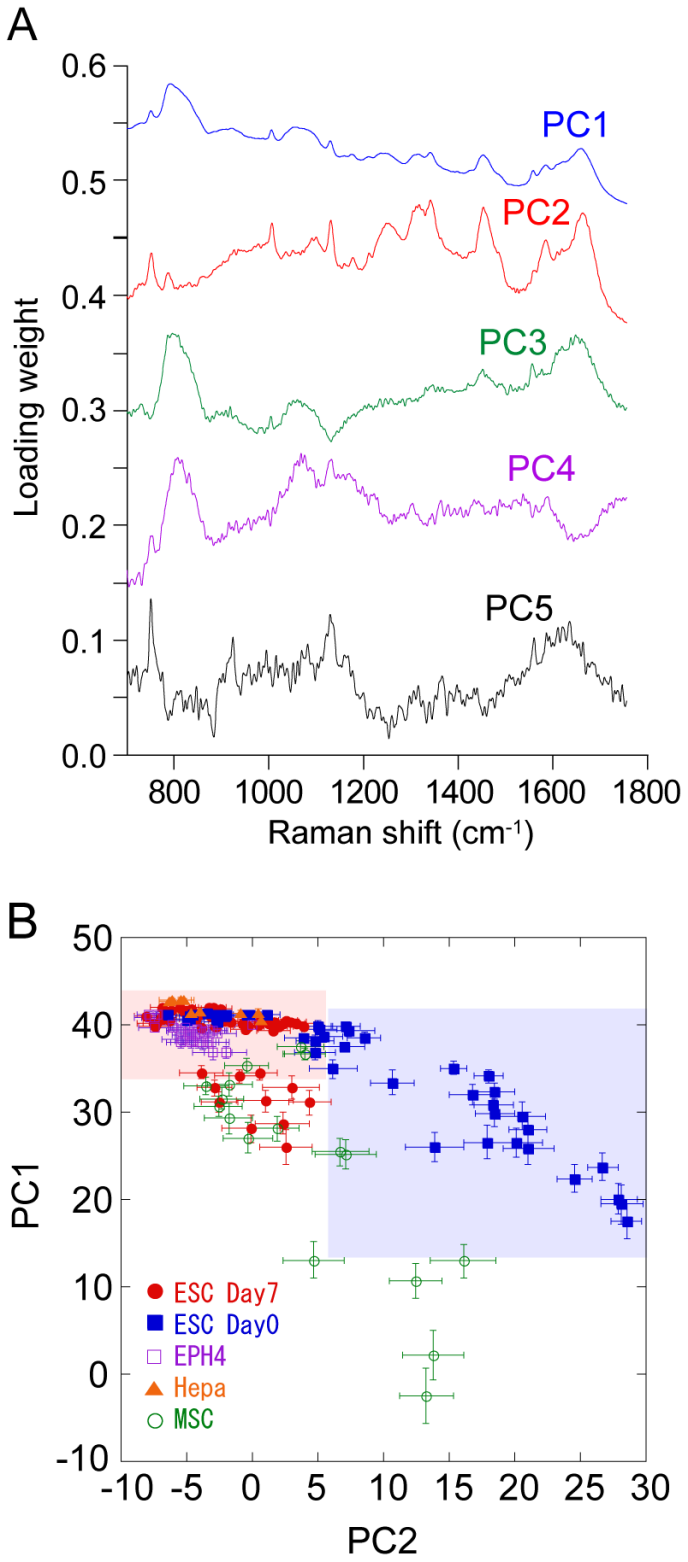
PCA analysis of Raman spectra obtained from ESCs. (A) Calculated loading vectors of PC1–PC5 of the Raman spectra of the nuclei in the fingerprint region. For PCA analysis, raw spectra without averaging was used. (B) Plot of PC1 and PC2 scores. Each marker shows the average value of the PC1 and PC2 scores of the Raman spectra obtained from single nuclei. Error bars show SD of the score values from the same nuclei.

Figure 4C shows averaged Raman spectra of the fingerprint region of the nucleus. The averaged Raman spectrum of the nucleus of MSCs was used as a model for the intermediate differentiation state in terms of differentiation capability. The Raman spectra of the MSCs are quite similar to those of the ESCs. PCA was performed against Raman spectra of ESCs before and after the induction of differentiation together with the cell-lines. Figure 5A represents the calculated loading vectors for the first five principal components (PC1–PC5). While PC3 and PC4 seemed predominated by the spectral features of silica (Fig. S1), PC1, PC2, and PC5 were mainly representative of the Raman spectra of the cells (Fig. 5A). Fig. 5B shows the score plot of PC1 and PC2 for ESCs, MSCs, EPH4 and Hepa1-6, with each dot corresponding to one nucleus and error bars representing the standard deviation (SD) of the score values of the nuclei. It is evident that the undifferentiated ESCs are widely distributed while the differentiated ESCs are localized in a different region of the PC1-PC2 plane, although there is some overlap. Moreover, MSCs lie between differentiated and undifferentiated ESCs, while EPH4 and Hepa1-6 cells are confined to a different region and slightly overlap with the differentiated ESCs.

### Fluctuation analysis of ESC differentiation by using PCA

**Figure 6:**
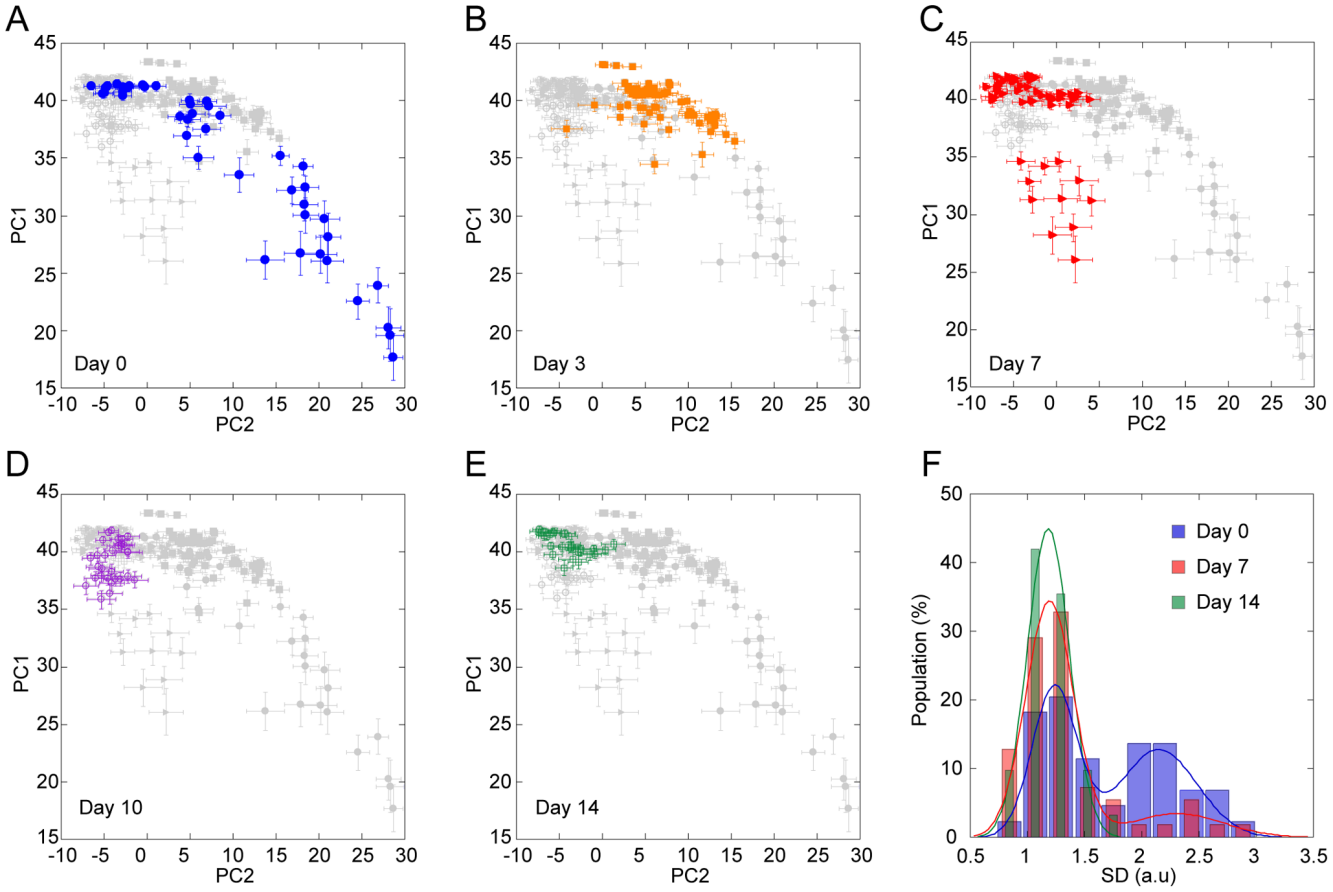
Transition of ESC state during differentiation. (A–E) Score plots of Raman spectra at various stages of differentiation spanning a period of 2 weeks. Each marker shows averaged score values of the spectra obtained from single nuclei. Error bar shows SD of the score values from the same nuclei. (F) Histograms of the population distribution of the SD relative to PC1 and PC2 before (blue), 1 week (red) and 2 weeks (green) after LIF removal. Solid lines show the results of the fitting achieved with two Gaussian functions.

To gain detailed information on the process of spontaneous ESC differentiation, we carried out a series of experiments that lasted 2 weeks (Fig. 6) and performed PCA for a dataset containing all the spectral data from Days 0, 3, 7, 10, and 14, with the data used from Day 0 and Day 7 being the same as those in Fig. 5. The calculated loading vectors were very similar to those used in Fig. 5, especially regarding PC1, PC2, and PC3 (Fig. S3). The results clearly illustrate a gradual transition of the cell population to the ‘differentiated’ region on the PC1-PC2 plane, with the distribution becoming narrower upon differentiation progression. Furthermore, the SD of the weights of PC1 and PC2 relative to the same nuclei, which represent the diversity among the nuclei, became smaller as differentiation progressed (see error bars in Fig. 6A–E). To quantify this observation, we built histograms describing the population distribution of the SD of PC1 and PC2 (Fig. 6F). The histograms relative to Day 0, 7, and 14 were normalized against the total number of observations. Fig. 6F shows that there are two distinct peaks only in the earlier stages (Day 0 and 7), and thus the larger SD population can be assigned to the undifferentiated cells. After ESC differentiation, the peak with the larger SD decreased while that with the smaller SD increased, suggesting that the smaller SD population should correspond to the differentiated cells. The larger SD population seen in the undifferentiated ESCs is localized in the low-PC1/high-PC2 region on the PC1-PC2 plane, indicating that the cells distributed in this region are ‘real’ undifferentiated cells. Interestingly, after the distribution of the low-PC1/high-PC2 population diminished at Day 3, another population of cells with a large distribution emerged in a site different from the initial population. Thus, it seems likely that Raman imaging can identify undifferentiated ESCs in heterogeneous cultures such as those arising from the current cell culture protocol. Moreover, we believe that the data shown in Fig. 6 reveal the appearance of a potential attractor at the initial stage of ESC differentiation.

## Discussion

In this study, we have succeeded in visualizing the cell-state transition during differentiation both in cell-lines and in ESCs. Based on these results, we suggest that there are substantial differences in the Raman spectra of the nuclei of differentiated and undifferentiated cells. Since it is well established that large changes in the epigenetic states occur during cell differentiation [22], changes in the Raman spectra of the nuclei may reflect these epigenetic changes. Although the origin of the difference in the Raman spectra of differentiated and undifferentiated cells is unclear, it is obvious that the differentiation state can be monitored using this technique. As previously stated, the undifferentiated and differentiated ESCs are located in different regions of the score plot (Fig. 5B) with some overlap. Since the established cell-lines are localized in the region delimited by the red rectangle in Fig. 5B, we can assume that undifferentiated ESCs positioned in this region are actually differentiated ESCs. Since MSCs are confined in the region between undifferentiated (blue rectangle in Fig. 5B) and differentiated ESCs, where a small population of differentiated ESCs is also positioned, we conclude that the time period of seven days after the removal of LIF was not long enough to induce the differentiation of all the ESCs, leaving them in an intermediate state between the undifferentiated and differentiated states.

Raman spectral difference between undifferentiated and differentiated cells were smaller in cell-lines with differentiation capability (N2a and 3T3L1) compared with ESCs. Judging from the differentiation capability, it can be expected that cell state differences are smaller in cell-lines with limited differentiation capability compared with pluripotent ESCs, which may reflect the small separation seen in the PCA analysis (Fig. 3C and 3F). In both cell-lines with differentiation capability, there were some overlaps between undifferentiated and differentiated cells, indicating that some cells remain in the undifferentiated state or vice versa. This speculation originates from the observation that some of the undifferentiated N2a cells extend neurites, and some of the differentiated 3T3L1 cells fail to accumulate lipid droplets. It is interesting to note that, even though 3T3L1 cells are sub-clones of NIH3T3 cells, the latter were located in an area of the PC1-PC2 plane different from 3T3L1. In particular, NIH3T3 cells widely spread in the PC1-PC2 plane with some overlap with undifferentiated and differentiated 3T3L1 cells. It is likely that NIH3T3 cells have a heterogeneous population, with some cells still possessing characteristics that are close to 3T3L1 cells. In conclusion, we demonstrated that the combination of Raman imaging and PCA is able to distinguish the cell state changes during differentiation.

The biological meaning of the Raman spectrum diversity within a nucleus is still unclear. It is well established that chromosomes are arranged in tightly packed heterochromatin-rich and loosely packed euchromatin-rich regions, which rearrange during differentiation [23], [24]. In addition, other particles such as Cajal bodies, nuclear speckles, nucleolus, and PML bodies are also present in the nucleus, with each occupying different regions [25], [26], [27]. These distinct nuclear sub-compartments consist of unique sets of resident proteins and specific functions. Many lines of evidence suggest that these nuclear domains undergo reorganization during cell differentiation [28], [29]. Since the nuclear sub-compartments in undifferentiated cells are poorly defined [30], it seems reasonable that the diversity of the Raman spectra of the nuclei may reflect the presence of these sub-compartments. The present study strongly suggests that Raman spectra enable the description of the cell state transition and the appearance of an attractor in the differentiation potential landscape even without comprehensive analysis.

In this study, we were able to distinguish the extent of differentiation of ESCs with the aid of Raman scattering microscopy combined with PCA, which we believe will be an advantageous tool to perform quality control on stem cells. Raman scattering carries information on all the constituents of a cell such as nucleic acids, proteins, and lipids, and the corresponding spectra are subject to changes in the concentration of those constituents. We think that morphology-based analysis of Raman spectra, rather than chemical or biological investigations, is sufficient to distinguish the cell status for medical and clinical applications. In this sense, Raman spectra can be used as a cellular fingerprint. In this context, PCA plays a role in pattern recognition rather than spectral analysis. By recording the spectra of known cell states, we aim to build a database of Raman spectra that can be compared with the spectra of unknown states of other cells. This concept is essentially analogous to the common practice in cell biology of recognizing and predicting the state of a cell by its morphological shape. Because Raman spectra contain a huge amount of information arising from the molecules inside the cell, Raman spectral morphology will potentially become an indicator for determining the state of the cells.

The present method, which treats Raman spectrum as a fingerprint of the cell, obtains only one phenotype of the cells. More comprehensive analysis, such as DNA microarray, enables to directly show the gene regulatory network, even though is not applicable for single cell analysis. Thus, in future work we aim to use our method to study the dynamics of attractors on not PC axes but in real axes, i.e., Oct4, Sox2 and Nanog in the stem cell state at a single cell level.

## Supporting Information

Figure S1 Averaged Raman spectra of the area without cells. 100 points from 25 individual experiments were averaged.
(TIF)

Figure S2 Phase contrast images of (A, B) Neuro2a and (C, D) 3T3L1 cells before (A, C) and after (B, D) induction of differentiation. Scale bar, 50 µm.
(TIF)

Figure S3 Calculated loading vectors of PC1∼PC5 used for PCA analysis in Fig. 6.
(TIF)
