# Supplementary figures and images for: Visualizing Cell State Transition Using Raman Spectroscopy
Source: PLoS One. 2014 Jan 7;9(1):e84478. doi: 10.1371/journal.pone.0084478 (PMC3883674; doi:10.1371/journal.pone.0084478)

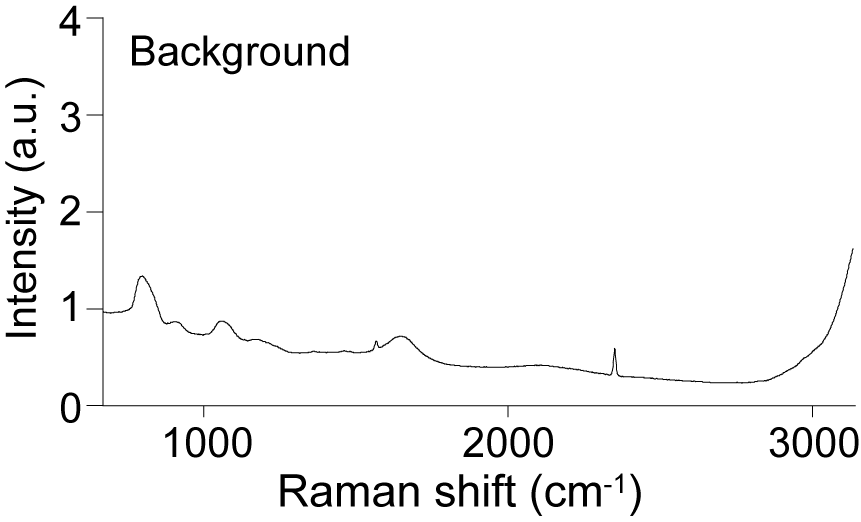

Supplement: Figure S1 — Averaged Raman spectra of the area without cells. 100 points from 25 individual experiments were averaged. (TIF) [file pone.0084478.s001.tif]

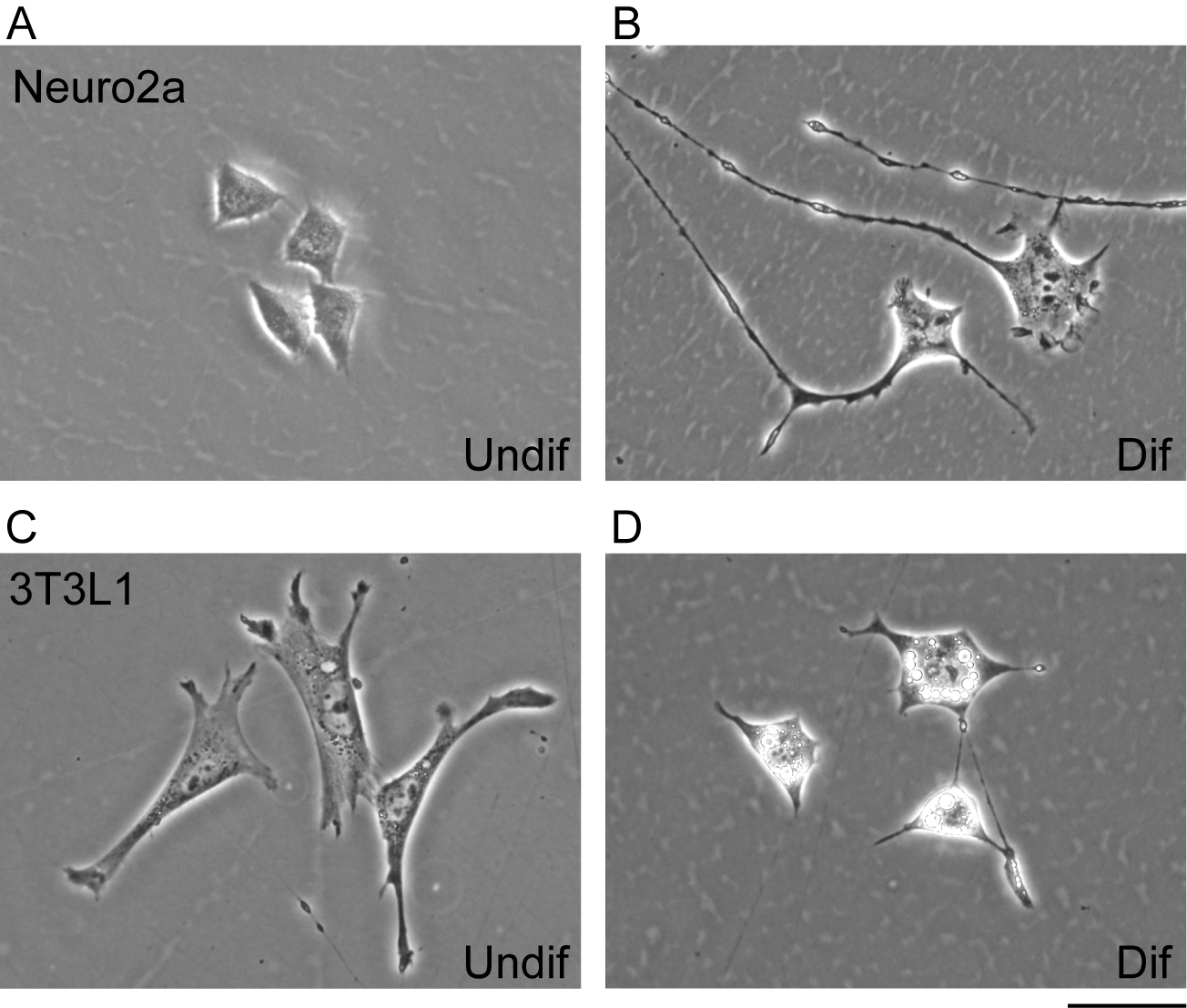

Supplement: Figure S2 — Phase contrast images of (A, B) Neuro2a and (C, D) 3T3L1 cells before (A, C) and after (B, D) induction of differentiation. Scale bar, 50 µm. (TIF) [file pone.0084478.s002.tif]

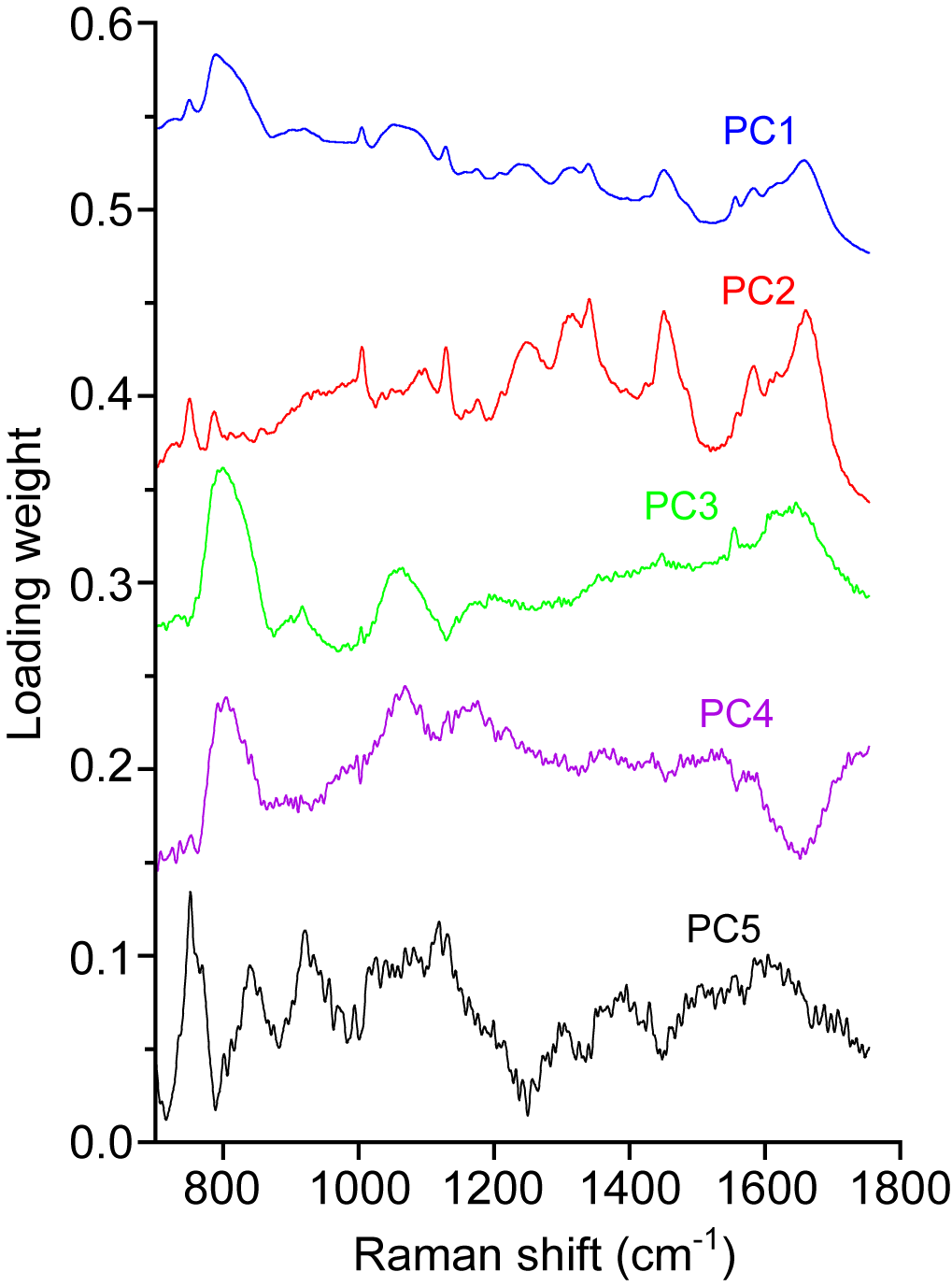

Supplement: Figure S3 — Calculated loading vectors of PC1∼PC5 used for PCA analysis in Fig. 6. (TIF) [file pone.0084478.s003.tif]
